# Role of Reactive Oxygen Species in the Progression of Type 2 Diabetes and Atherosclerosis

**DOI:** 10.1155/2010/453892

**Published:** 2010-02-16

**Authors:** Hideaki Kaneto, Naoto Katakami, Munehide Matsuhisa, Taka-aki Matsuoka

**Affiliations:** Department of Metabolic Medicine, Graduate School of Medicine, Osaka University, 2-2 Yamadaoka, Suita, Osaka 565-0871, Japan

## Abstract

Type 2 diabetes is the most prevalent and serious metabolic disease all over the world, and its hallmarks are pancreatic *β*-cell dysfunction and insulin resistance. Under diabetic conditions, chronic hyperglycemia and subsequent augmentation of reactive oxygen species (ROS) deteriorate *β*-cell function and increase insulin resistance which leads to the aggravation of type 2 diabetes. In addition, chronic hyperglycemia and ROS are also involved in the development of atherosclerosis which is often observed under diabetic conditions. Taken together, it is likely that ROS play an important role in the development of type 2 diabetes and atherosclerosis.

## 1. Augmentation of Reactive Oxygen Species (ROS) by Various Pathways under Diabetic Conditions

**Figure 1:**
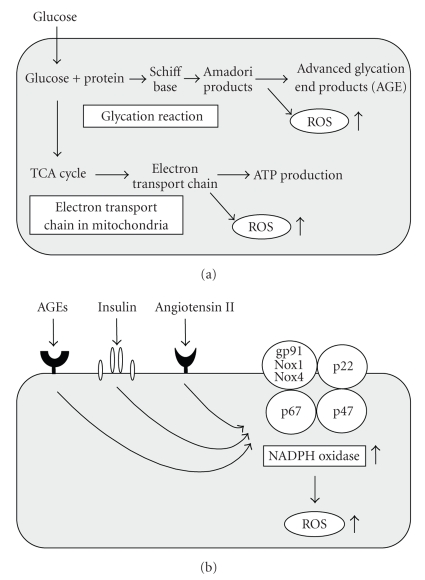
*Augmentation of ROS by various pathways under diabetic conditions.* (a) ROS are produced by various pathways under diabetic conditions. Hyperglycemia induces ROS through activation of the glycation reaction and electron transport chain in mitochondria. (b) AGEs, insulin, and angiotensin II induces ROS through activation of membrane-bound NADPH oxidase.

It has been shown that ROS are produced in various tissues under diabetic conditions [1, 2]. There are several sources of ROS in cells such as the nonenzymatic glycosylation reaction [3], the electron transport chain in mitochondria [4], and membrane-bound NADPH oxidase [5, 6]. In diabetic animals, glycation reaction is observed in various tissues and organs, and various kinds of glycated proteins such as glycosylated hemoglobin, albumin, and lens crystalline are produced in a nonenzymatical manner through the glycation reaction. The reaction produces Schiff base, Amadori product, and finally advanced glycosylation end products (AGEs). During the process, ROS are also produced. The electron transport chain in mitochondria is also an important pathway to produce ROS. Under diabetic conditions, electron transport chain is activated, which leads to production of larger amounts of ROS. It has been shown that membrane-bound NADPH oxidase is also an important source of ROS. NADPH oxidase is composed of the membrane-bound subunits gp91 phox (Nox2)/Nox1/Nox4 and p22 phox and the catalytic site of the oxidase and cytosolic components p47 phox and p67 phox. NADPH oxidase is activated by various stimuli such as AGEs, insulin, and angiotensin II; all of which are possibly induced under diabetic conditions (Figures 1(a) and 1(b)).

## 2. Role of ROS in the Progression of Pancreatic *β*-Cell Dysfunction in Type 2 Diabetes

Acute exposure of  *β*-cells to a high glucose concentration stimulates insulin gene expression, but chronic exposure has various adverse effects on various *β*-cell function. However, chronic hyperglycemia is a cause of impairment of insulin biosynthesis and secretion. This process is called *β*-cell glucose toxicity which is often observed under diabetic conditions. In the diabetic state, hyperglycemia per se and subsequent production of ROS decrease insulin gene expression and secretion and finally bring about apoptosis [7–28]. It has been shown that the loss of insulin gene expression is accompanied by decreased expression and/or DNA binding activities of transcription factors: pancreatic and duodenal homeobox-1 (PDX-1) [19–26] and MafA [10, 12, 15]. After chronic exposure to a high glucose concentration, expression and/or DNA binding activities of these two transcription factors are reduced. It is noted here that PDX-1 plays a crucial role in pancreas development, *β*-cell differentiation, induction of surrogate *β*-cells, and maintenance of mature *β*-cell function [29–41] and that MafA is a recently isolated *β*-cell-specific transcription factor and functions as a potent activator of insulin gene transcription [42–47]. 

Under diabetic conditions, ROS are induced and involved in the *β*-cell glucose toxicity [22–36]. *β*-Cells express GLUT2, a high-Km glucose transporter, and thereby display highly efficient glucose uptake when exposed to a high glucose concentration. Indeed, it was shown that expressions of ROS markers 8-hydroxy-2′-deoxyguanosine (8-OHdG) and 4-hydroxy-2, 3-nonenal (4-HNE) were increased in islets under diabetic conditions [14, 16]. In addition, *β*-cells are rather vulnerable to ROS due to the relatively low expression of antioxidant enzymes such as catalase and glutathione peroxidase [48, 49]. Therefore, it is likely that ROS are involved in *β*-cell deterioration found in diabetes. It was shown that when *β*-cell-derived cell lines or rat isolated islets were exposed to ROS, insulin gene promoter activity and mRNA expression were suppressed [19–26]. In addition, when they were exposed to ROS, binding of PDX-1 and/or MafA to the insulin gene promoter was markedly reduced. Furthermore, it was shown that the decrease of insulin gene expression after chronic exposure to a high glucose concentration was prevented by treatment with antioxidants [16, 19, 25, 26]. Reduction of expression and/or DNA binding activities of PDX-1 and MafA by chronic exposure to high glucose was also prevented by an antioxidant treatment. These results suggest that chronic hyperglycemia suppresses insulin biosynthesis and secretion by increasing ROS, accompanied by reduction of expression and/or DNA binding activities of two important pancreatic transcription factors: PDX-1 and MafA. Therefore, it is likely that the alteration of such transcription factors explains, at least in part, the suppression of insulin biosynthesis and secretion, and thus are involved in *β*-cell glucose toxicity. Indeed, it was shown that the antioxidant treatment with N-acetyl-L-cysteine plus vitamin C and E retained glucose-stimulated insulin secretion and moderately ameliorated glucose tolerance in obese diabetic C57BL/KsJ-db/db mice [19]. *β*-Cell mass was significantly larger in the mice treated with the antioxidants, and insulin content was preserved by the antioxidant treatment. Furthermore, PDX-1 expression was more clearly visible in the nuclei of *β*-cells after the antioxidant treatment [19]. Similar effects were observed with Zucker diabetic fatty rats, another model animal for type 2 diabetes [25]. Therefore, it is likely that antioxidant treatment can protect *β*-cells against glucose toxicity. In addition, angiotensin II type 1 receptor blocker (ARB) has antioxidant effects and thereby treatment with ARB exerts beneficial effects for diabetes [50, 51]. Indeed, it was shown that when diabetic C57BL/KsJ-db/db mice were treated with ARB, *β*-cell mass and insulin content were increased, and expressions of components of NAD(P)H oxidase and ROS markers in *β*-cells were decreased. The ARB treatment also reduced fibrosis in and around the islets and prevented the loss of endothelial cells in islets. These results suggest that ARB treatment protects *β*-cells by reducing ROS and further strengthen the hypothesis that ROS are involved in *β*-cell glucose toxicity found in diabetes.

It is known that lipotoxicity is also involved in the deterioration of *β*-cell function found in type 2 diabetes. When islets or *β*-cell-derived cell lines were exposed to free fatty acids (FFAs), ROS were induced, which led to the reduction of insulin secretion and *β*-cell dysfunction [52–56]. It was also reported that FFA-mediated induction of inducible nitric oxide synthase (iNOS) and excess nitric oxide (NO) generation were involved in the progression of *β*-cell dysfunction [57]. Because intracellular NO is an important mediator of *β*-cell apoptosis [58], it is likely that the loss of *β*-cell mass observed in type 2 diabetes is due to NO-induced apoptosis. 

**Figure 2:**
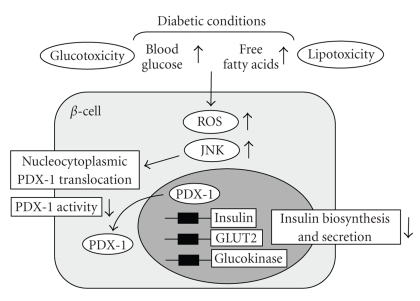
*Role of ROS in the progression of pancreatic* *β* *-cell dysfunction in type 2 diabetes.* ROS are provoked by hyperglycemia and/or hyperlipidemia under diabetic conditions, which leads to activation of the JNK pathway in pancreatic *β*-cells. ROS and subsequent activation of the JNK pathway induce nucleo-cytoplasmic translocation of PDX-1, which leads to reduction of PDX-1 activity and suppression of insulin. Therefore, it is likely that ROS and activation of the JNK pathway are involved in *β*-cell dysfunction found in type 2 diabetes.

It has been suggested that activation of the c-Jun N-terminal kinase (JNK) pathway is involved in pancreatic *β*-cell dysfunction found in type 2 diabetes. It was reported that activation of the JNK pathway is involved in reduction of insulin gene expression by ROS and that suppression of the JNK pathway can protect *β*-cells from ROS [59]. When isolated rat islets were exposed to ROS, the JNK pathway was activated, preceding the decrease of insulin gene expression. Adenoviral overexpression of dominant-negative type JNK1 (DN-JNK) protected insulin gene expression and secretion from ROS. These results were correlated with change in the binding of PDX-1 to the insulin promoter. Adenoviral overexpression of DN-JNK preserved PDX-1 DNA binding activity in the face of ROS, while WT-JNK overexpression decreased PDX-1 DNA binding activity [59]. Taken together, it is likely that activation of the JNK pathway leads to decreased PDX-1 activity and consequent suppression of insulin gene transcription found in the diabetic state. Also, it was shown that PDX-1 is translocated from the nuclei to the cytoplasm in response to ROS. When *β*-cell-derived HIT cells were exposed to ROS, both intrinsically expressed PDX-1 and exogenously introduced green fluorescent protein (GFP)-tagged PDX-1 moved from the nuclei to the cytoplasm [60]. DN-JNK overexpression inhibited the ROS-induced PDX-1 translocation, suggesting that activation of the JNK pathway is involved in PDX-1 translocation by ROS. Furthermore, leptomycin B, a specific inhibitor of the classical, leucine-rich nuclear export signal (NES), inhibited nucleo-cytoplasmic translocation of PDX-1 induced by ROS [60]. Taken together, it is likely that ROS induce nucleo-cytoplasmic translocation of PDX-1 through activation of the JNK pathway, which leads to reduction of its DNA binding activity and suppression of insulin biosynthesis (Figure 2).

The forkhead transcription factor Foxo1 is known as one of the important fundamental transcription factors playing a key role in apoptosis, cellular proliferation and differentiation, and glucose metabolism through regulating the transcription of various target genes [61, 62]. It was shown that Foxo1 regulates hepatic gluconeogenesis, and thus contributes to insulin resistance [63]. Insulin inhibits the function of Foxo1 through Akt/PKB-mediated phosphorylation and nuclear exclusion [64], and thereby suppresses hepatic gluconeogenesis. It was also shown that Foxo1 exhibits a counter localization to PDX-1 in *β*-cells [65], suggesting that it is involved in the deterioration of *β*-cell function. Moreover, it was shown that Foxo1 plays a role as a mediator between the JNK pathway and PDX-1 [66]. In *β*-cell-derived cell line HIT-T15, Foxo1 changed its intracellular localization from the cytoplasm to the nucleus after exposure to ROS. In contrast to Foxo1, the nuclear expression of PDX-1 was decreased and its cytoplasmic distribution was increased by ROS. Activation of the JNK pathway also induced the nuclear localization of Foxo1, whereas suppression of the JNK pathway reduced the ROS-induced nuclear localization of Foxo1, suggesting an involvement of the JNK pathway in Foxo1 translocation [66]. In addition, ROS or activation of the JNK pathway decreased Akt phosphorylation in HIT cells, leading to the decreased phosphorylation of Foxo1 following nuclear localization. Furthermore, adenoviral Foxo1 overexpression reduced the nuclear expression of PDX-1, whereas suppression of Foxo1 by Foxo1-specific small interfering RNA retained the nuclear expression of PDX-1 [66]. Taken together, ROS and subsequent activation of the JNK pathway induce nuclear translocation of Foxo1 through the modification of the insulin signaling in *β*-cells, which leads to the nucleo-cytoplasmic translocation of PDX-1 and reduction of its DNA binding activity. It was also shown that the mammalian Ste20-like kinase 1 (MST1) is activated by ROS, which facilitates Foxo1 translocation from the cytoplasm to the nuclei [67]. Therefore, it is also possible that ROS trigger Foxo1 translocation from the cytoplasm to the nuclei, independently of Akt activity or Akt-mediated phosphorylation status of Foxo1. Furthermore, the significance of the JNK pathway in the development of diabetes comes from the result of a genetic analysis in humans. While islet-brain-1 (IB1) was known to suppress the JNK pathway [68, 69], it was shown that a missense mutation within the IB1-encoding MAPKIP1 gene (S59N) is associated with a late onset type 2 diabetes [70]. Thus, it is likely that activation of the JNK pathway is involved in deterioration of *β*-cell function found in type 2 diabetes.

## 3. Role of ROS in the Progression of Insulin Resistance in Type 2 Diabetes

The hallmark of type 2 diabetes is insulin resistance as well as pancreatic *β*-cell dysfunction. Under diabetic conditions, various insulin target tissues such as the liver, muscle, and fat become resistant to insulin. The pathophysiology of insulin resistance involves a complex network of insulin signaling pathways. After insulin binds to insulin receptor on cell surface, insulin receptor and its substrates are phosphorylated, which leads to activation of various insulin signaling pathways [71–74]. It has been shown that ROS are involved in the progression of insulin resistance as well as pancreatic *β*-cell dysfunction [75]. Indeed, it was previously reported that ROS disrupted insulin-induced cellular redistribution of insulin receptor substrate-1 (IRS-1) and phosphatidylinositol 3-kinase (PI 3-K), and thus impaired insulin-induced GLUT4 translocation in 3T3-L1 adipocyte [76, 77]. It was also reported that treatment with antioxidants (N-acetyl-L-cysteine and taurine) prevented hyperglycemia-induced insulin resistance in vivo [78]. Furthermore, in patients with type 2 diabetes, both acute and chronic administrations of *α*-lipoic acid, an antioxidant, improved insulin resistance, suggesting that ROS are involved in the progression of insulin resistance [79, 80].

**Figure 3:**
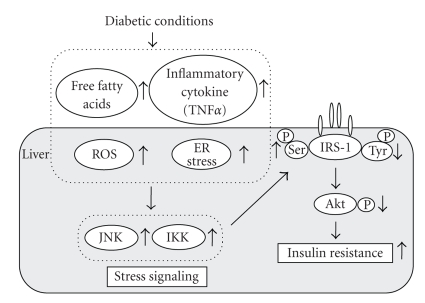
*Role of ROS in the development of insulin resistance in type 2 diabetes.* The JNK pathway is activated by various factors such as ROS, ER stress, free fatty acids (FFAs), and inflammatory cytokines such as TNF*α* and is involved in the development of insulin resistance found in type 2 diabetes. It has been also shown that the IkappaB kinase *β* (IKK) pathway is also activated by such factors and is involved in the development of insulin resistance. Therefore, it is likely that activation of stress signaling is involved in the development of insulin resistance.

Under diabetic conditions, hyperglycemia increased ROS, which presumably lead to activation of the JNK pathway. In addition, under diabetic conditions, free fatty acids (FFAs), various inflammatory cytokines (e.g., TNF*α*), and endoplasmic reticulum (ER) stress are increased, which also leads to activation of the JNK pathway. Finally, it has been suggested that activation of the JNK pathway is involved in insulin resistance as well as pancreatic *β*-cell dysfunction found in diabetes [81, 82]. It was reported that the JNK pathway was abnormally activated in the liver, muscle, and adipose tissue in obese type 2 diabetic mice and that insulin resistance in obese type 2 diabetic mice was substantially reduced in mice homozygous for a targeted mutation in the JNK1 gene (JNK-KO mice) [83]. When the JNK-KO and control mice were placed on a high-fat/high-caloric diet, blood glucose levels in the obese JNK-KO mice were significantly lower compared to obese wild-type mice. Intraperitoneal insulin tolerance tests showed that hypoglycemic response to insulin in obese wild-type mice was lower compared to obese JNK-KO mice. Also, intraperitoneal glucose tolerance test revealed a higher degree of hyperglycemia in obese wild-type mice than obese JNK-KO mice. These results indicate that the JNK-KO mice are protected from the development of dietary obesity-induced insulin resistance. Furthermore, targeted mutations in JNK1 were introduced in genetically obese mice (ob/ob) [83]. Blood glucose levels in the ob/ob-JNK-KO mice were lower compared to ob/ob wild-type mice, and the ob/ob wild-type mice displayed a severe and progressive hyperinsulinemia. Therefore, it is likely that JNK1 deficiency provides resistance against obesity, hyperglycemia, and hyperinsulinemia in both genetic and dietary models of diabetes. These results suggest that activation of the JNK pathway plays an important role in the development of insulin resistance found in type 2 diabetes. It was also reported that overexpression of dominant-negative (DN) type JNK1 (Ad-DN-JNK) in the liver of obese diabetic C57BL/KsJ-db/db mice improved insulin resistance and ameliorated glucose intolerance [83]. In intraperitoneal insulin tolerance test, the hypoglycemic response to insulin was larger in Ad-DN-JNK-treated db/db mice. Furthermore, in the euglycemic hyperinsulinemic clamp test, glucose infusion rate (GIR) in Ad-DN-JNK-treated mice was higher than that in Ad-GFP-treated mice, indicating that suppression of the JNK pathway in the liver reduces insulin resistance, and thus ameliorates glucose intolerance in the db/db mice. Furthermore, hepatic glucose production (HGP) was significantly lower in Ad-DN-JNK-treated mice, whereas there was no difference in the glucose disappearance rate (Rd) between these two groups [84]. These results indicate that reduction of insulin resistance and amelioration of glucose tolerance by DN-JNK overexpression are mainly due to suppression of hepatic glucose production. It has been reported that serine phosphorylation of insulin receptor substrate-1 (IRS-1) inhibits insulin-stimulated tyrosine phosphorylation of IRS-1, leading to an increase in insulin resistance [85]. IRS-1 serine 307 phosphorylation was decreased and IRS-1 tyrosine phosphorylation was increased in Ad-DN-JNK-treated mice [84]. Therefore, it is likely that an increase in IRS-1 serine phosphorylation is associated with the development of insulin resistance induced by JNK overexpression. Taken together, suppression of the JNK pathway enhances insulin signaling which leads to amelioration of glucose tolerance (Figure 3).

Protein transduction domains (PTDs) such as the small PTD from the TAT protein of human immunodeficiency virus (HIV-1), the VP22 protein of Herpes simplex virus, and the third *α*-helix of the homeodomain of Antennapedia, a Drosophila transcription factor, are known to allow various proteins and peptides to be efficiently delivered into cells through the plasma membrane, and thus there has been increasing interest in their potential usefulness for the delivery of bioactive proteins and peptides into cells [86–91]. It was reported that the cell permeable JNK inhibitory peptide is effective for the treatment of diabetes. This peptide is derived from the JNK binding domain of JNK-interacting protein-1 (JIP-1) and has been reported to function as a dominant inhibitor of the JNK pathway [92]. It is noted here that JIP-1 itself is a scaffold protein which binds JNK and activates the JNK pathway. When this peptide was injected intraperitoneally to C57BL/KsJ-db/db obese diabetic mice, the FITC-conjugated peptide showed fluorescence signals in insulin target organs (liver, fat, and muscle) and in insulin secreting tissue (pancreatic islets) [93]. In insulin tolerance test, reduction of blood glucose levels in response to injected insulin was larger in JNK inhibitory peptide-treated mice [93]. Furthermore, in the euglycemic hyperinsulinemic clamp test, the steady-state glucose infusion rate (GIR) in JNK inhibitory peptide-treated mice was higher than that in untreated mice, indicating that JNK inhibitory peptide reduces insulin resistance in the db/db mice. Endogenous hepatic glucose production (HGP) and glucose disappearance rate (Rd) in the JNK inhibitory peptide-treated mice were also evaluated. It is noted that Rd reflects glucose utilization in the peripheral tissues. HGP in JNK inhibitory peptide-treated mice was lower than that in untreated mice. In addition, Rd in JNK inhibitory peptide-treated mice was higher than that in untreated mice [93]. These results indicate that JNK inhibitory peptide treatment reduces insulin resistance through decreasing HGP and increasing Rd. IRS-1 serine 307 phosphorylation was decreased and IRS-1 tyrosine phosphorylation was increased in the peptide-treated mice. Concomitantly, glucose intolerance was also ameliorated in JNK inhibitory peptide-treated mice. Taken together, suppression of the JNK pathway improves insulin resistance and ameliorates glucose intolerance, which further strengthens the significance of the JNK pathway in the development of insulin resistance.

The JNK pathway is activated by various factors including ROS, ER stress, FFAs, and inflammatory cytokines such as TNF*α* and is involved in the development of insulin resistance found in type 2 diabetes [94–96]. It has been shown the IkappaB kinase *β* (IKK) pathway is also activated by such factors and is involved in the development of insulin resistance [97–100]. Activation of the IKK pathway increases IRS-1 serine phosphorylation which leads to suppression of insulin signaling. Also, suppression of the IKK pathway decreases insulin resistance and ameliorates glucose intolerance in diabetic mice. Therefore, it is likely that activation of stress signaling such as the JNK and IKK pathways is involved in the development of insulin resistance and that such pathways could be a therapeutic target for diabetes (Figure 3).

## 4. Role of ROS in the Progression of Atherosclerosis

Atherosclerosis is often observed as a macroangiopathy under diabetic conditions. Indeed, it has been reported that increase of intima-media thickness (IMT) in carotid artery, an index of the progression of atherosclerosis, is often observed in diabetic patients [101–103] and that the progression of IMT is influenced by a variety of genetic risk factors [104–106] and/or intervention for diabetes [107–109]. It is well known that hyperglycemia per se found under diabetic conditions facilitates the progression of atherosclerosis. In addition, hyperinsulinemia which is often observed in subjects with insulin resistance is likely involved in the progression of atherosclerosis. 

**Figure 4:**
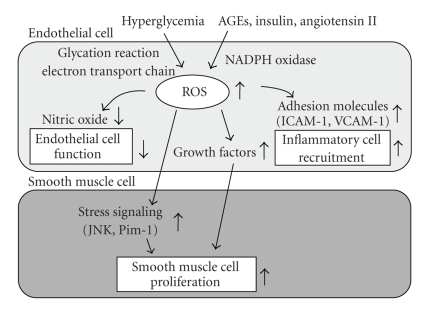
*Role of ROS in the development of atherosclerosis.* ROS are produced by various pathways under diabetic conditions and involved in the development of atherosclerosis in various aspects. Hyperglycemia induces ROS through activation of the glycation reaction and electron transport chain in mitochondria. Also, AGEs, insulin, and angiotensin II induce ROS through activation of NADPH oxidase. Increased ROS are involved in the development of atherosclerosis in various aspects. First, ROS decrease nitric oxide levels, which leads to endothelial cell dysfunction. Second, ROS increase expression of various adhesion molecules such as ICAM-1 and VCAM-1, which leads to inflammatory cell recruitment. Finally, ROS increase expression of various growth factors and activate various stress signaling such as JNK and Pim-1, which leads to proliferation of smooth muscle cell.

It has been shown that ROS are induced in endothelial cells under diabetic conditions. There are several sources of reactive oxygen species (ROS) in cells such as the nonenzymatic glycosylation reaction, the electron transport chain in mitochondria, and membrane-bound NADPH oxidase (Figure 4). It has been shown that membrane-bound NADPH oxidase is the one of the major sources of ROS in the vasculature and that NADPH oxidase-derived ROS play a critical role in the development of atherosclerosis. NADPH oxidase is composed of the membrane-bound subunits gp91 phox (Nox2)/Nox1/Nox4 and p22 phox, and the catalytic site of the oxidase and cytosolic components p47 phox and p67 phox. In vascular cells such as endothelial and smooth muscle cells, Nox 1 and Nox 4, rather than gp91 phox, are abundantly expressed. NADPH oxidase is activated by various factors such as AGEs, insulin, and angiotensin II; all of which are possibly induced under diabetic conditions [110]. In addition, it was shown that high glucose stimulates ROS production through the activation of NADPH oxidase [111, 112] and that the p22 phox was significantly increased in rat and human diabetic arteries [113, 114]. Therefore, it is possible that such increased expression of p22 phox contributes to the development of atherosclerosis. It was also reported that mice lacking p47 phox, which is an important component for NADPH oxidase, had lower levels of aortic ROS production compared with wild-type mice and that when the mice were crossed with apolipoprotein E knockout (p47 phox (−/−), apoE (−/−)) mice they had significantly fewer lesions in their descending aortas compared to p47 phox (+/+), apoE (−/−) mice [115]. NADPH oxidase-derived ROS play a crucial role in the development of atherosclerosis in human as well as in mice. Indeed, it has been reported that ROS production in atherosclerotic human coronary arteries is associated with NADPH oxidase subunit p22 phox [116]. Also, it has been reported recently that phagocytic NADPH oxidase overactivity is involved in ROS and atherosclerosis in metabolic syndrome patients and that hyperinsulinemia likely contributes to ROS in metabolic syndrome patients through activation of NADPH oxidase [117]. 

In addition, it is likely that the vulnerability to oxidative stress is determined by genetic background. There are several enzymes regulating redox status and the vulnerability to oxidative stress is affected by genetic polymorphisms in these enzymes. For example, it was reported that in type 2 diabetic subjects the C242T polymorphism of the p22 phox gene, an essential component of NADPH oxidase in the vasculature, was closely associated with intima-media thickness (IMT) of the carotid artery, an index of the progression of atherosclerosis [105]. It is noted here that the presence of 242T allele is known to be associated with significantly reduced vascular NADPH oxidase activity. Average IMT in the diabetic subjects with the CC genotype was significantly higher compared to those with the TC + TT genotypes. Furthermore, in stepwise multiple regression analysis, p22 phox CC genotype was an independent risk factor for increased IMT in the diabetic subjects [105]. These results suggest that the vulnerability to oxidative stress and the progression of atherosclerosis are influenced by genetic background. Furthermore, the accumulation of oxidative stress-associated gene polymorphisms is likely associated with the severity and the progression of atherosclerosis in diabetic patients. For example, it was reported that carotid intima-media thickness (IMT) as well as serum 8-OHdG level, a marker of oxidative stress, were closely associated with the accumulation of several oxidative stress-associated gene polymorphisms, such as the T allele of the C-588T polymorphism in glutamate-cysteine ligase modifier subunit (GCLM) gene, the GG genotype of the G-463A polymorphism in myeloperoxidase (MPO) gene, the substitution of Gln for Arg at position 192 in human paraoxonase (PON1), and the T allele of the C242T polymorphism in NAD(P)H oxidase p22 phox gene [118]. Furthermore, the accumulation of these 4-gene polymorphisms was closely associated with the progression of carotid IMT in the longitudinal settings. In a stepwise multivariate regression analysis, the number of prooxidant alleles was an independent risk factor for the progression of IMT [119]. These results further support the hypothesis that the vulnerability to oxidative stress and the progression of atherosclerosis are influenced by genetic background. Furthermore, it was reported that the prevalence of myocardial infarction was significantly higher in the subjects with higher number of prooxidant alleles of these 4 gene polymorphisms [120]. Therefore, it is likely that the accumulation of oxidative stress-related gene polymorphisms influences the prevalence of myocardial infarction as well as atherosclerosis. 

Increased ROS are involved in the development of atherosclerosis in various aspects. First, endothelial dysfunction is an early key event in atherosclerosis [121–123]. It has been thought that ROS are involved in the progression of endothelial cell dysfunction, which is accompanied by inactivation of endothelial nitric oxide synthase (eNOS) and decrease of nitric oxide (NO) levels [124]. Second, ROS also induce expression of adhesion molecules such as intercellular adhesion molecule-1 (ICAM-1) and vascular adhesion molecule-1 (VCAM-1), which facilitates inflammatory cell recruitment and lipid deposition in the intimal layer. The subsequent ingestion of excess oxidized low density lipoprotein (LDL) particles by macrophages and monocytes leads to release of various inflammatory cytokines and growth factors. Finally, proliferation of vascular smooth muscle cells (VSMCs) is a key step in the development of atherosclerosis. It has been suggested that ROS regulate expression of various growth factors and several growth-related protooncogenes such as c-Myc, c-Fos and c-Jun [124, 125]. Clinical mass studies have also provided support for the significance of ROS in the development of atherosclerosis [126, 127]. Taken together, it is likely that ROS are involved in the VSMC proliferation and development of atherosclerosis through various pathways (Figure 4).

The JNK pathway is known to be activated by ROS in VSMC [128], and activation of the JNK pathway is likely involved in the progression of atherosclerosis. It is known that the JNK pathway plays an important role in the initiation of cellular responses, including cellular gene expression, growth, migration, or apoptosis. It has been previously reported that the JNK pathway is activated in balloon-injured arteries as well [129–131]. In vivo transfection of DN-JNK significantly suppressed activation of the JNK pathway and reduced VSMC proliferation in a balloon-injury model [132]. Neointimal formation after balloon-injury was also prevented by DN-JNK overexpression. Bromodeoxyuridine labeling index and total cell-counting analysis showed that DN-JNK remarkably suppressed VSMC proliferation in both the intima and the media after injury. In contrast, gene transfer of wild-type JNK (WT-JNK) significantly enhanced neointimal hyperplasia after balloon-injury. Taken together, activation of the JNK pathway triggers VSMC proliferation, leading to neointimal formation, and the JNK pathway could be a therapeutic target for atherosclerosis. The role of JNK in atherosclerotic plaque formation in vivo was also examined using atherosclerosis-prone apolipoprotein E knockout mice (ApoE (−/−) mice). Activation of the JNK pathway was closely correlated with the presence of clearly established plaques in ApoE (−/−) mice with a high-cholesterol diet. It was recently reported that atherosclerosis-prone ApoE (−/−) mice simultaneously lacking JNK2 (ApoE (−/−), JNK2 (−/−) mice) developed less atherosclerosis compared to ApoE (−/−) mice [133]. Pharmacological inhibition of the JNK activity also efficiently reduced plaque formation [134]. Macrophages lacking JNK2 displayed suppressed foam cell formation caused by defective uptake and degradation of modified lipoproteins and showed increased amounts of the modified lipoprotein-binding and -internalizing scavenger receptor A (SR-A). Macrophage-restricted deletion of JNK2 was sufficient to decrease atherogenesis [133]. These data suggest that JNK2-dependent phosphorylation of SR-A promotes uptake of lipids in macrophages, and thereby regulates foam cell formation. These results strengthen the significance of the JNK pathway in the progression of atherosclerosis. Furthermore, it was shown that Pim-1, a protooncogene that encodes a serine/threonine kinase, is also induced by ROS, and thus is likely involved in the progression of atherosclerosis [135, 136]. Pim-1 was substantially induced in neointimal VSMC of balloon injured rat carotid arteries, and in vivo infection with a dominant-negative Pim-1-expressing adenovirus (Ad-DN-Pim-1) markedly suppressed neointima formation and cell cycle progression in the balloon injured arteries [135]. In cultured VSMC, ROS-stimulated cell cycle progression and DNA synthesis were suppressed by DN-Pim-1 overexpression. Furthermore, Pim-1-producing cells were observed predominantly in the thickened intima of human thoratic aortas and coronary arteries [135]. These findings suggest that ROS and consequent induction of Pim-1 expression also play an important role in the progression of atherosclerosis. Taken together, ROS and subsequent activation of various stress signaling such as JNK and Pim-1 are involved in the progression of atherosclerosis (Figure 4).

## 5. Conclusion

ROS are induced under diabetic conditions, which are possibly involved in the progression of pancreatic *β*-cell dysfunction and insulin resistance found in type 2 diabetes. Suppression of ROS in obese type 2 diabetic mice restores *β*-cell function and insulin sensitivity, leading to amelioration of glucose intolerance. In addition, ROS are involved in the progression of atherosclerosis which is often observed as a macroangiopathy under diabetic conditions. Taken together, it is likely that ROS are closely associated with the development of type 2 diabetes and atherosclerosis. Although at present several clinical trials with antioxidants show only a little effect, if any, on the progression of type 2 diabetes, we think that future therapy with stronger and more appropriate antioxidants would exert some beneficial effects on the development of type 2 diabetes and atherosclerosis.
